# Induction of humoral and cell-mediated immunity to the NS1 protein of TBEV with recombinant Influenza virus and MVA affords partial protection against lethal TBEV infection in mice

**DOI:** 10.3389/fimmu.2023.1177324

**Published:** 2023-07-07

**Authors:** Jana Beicht, Mareike Kubinski, Isabel Zdora, Christina Puff, Jeannine Biermann, Thomas Gerlach, Wolfgang Baumgärtner, Gerd Sutter, Albert D. M. E. Osterhaus, Chittappen Kandiyil Prajeeth, Guus F. Rimmelzwaan

**Affiliations:** ^1^ Research Center for Emerging Infections and Zoonoses, University of Veterinary Medicine Hannover, Foundation, Hannover, Germany; ^2^ Department of Pathology, University of Veterinary Medicine Hannover, Foundation, Hannover, Germany; ^3^ Center for Systems Neuroscience, Hannover Graduate School for Neurosciences, Infection Medicine, and Veterinary Sciences (HGNI), Hannover, Germany; ^4^ Division of Virology, Institute for Infectious Diseases and Zoonoses, Ludwig Maximilian University (LMU) Munich, Munich, Germany; ^5^ German Center for Infection Research (DZIF), Partner Site Munich, Munich, Germany

**Keywords:** TBEV, IAV, MVA, NS1, protection, vaccination, virus-neutralizing antibodies, T cells

## Abstract

**Introduction:**

Tick-borne encephalitis virus (TBEV) is one of the most relevant tick-transmitted neurotropic arboviruses in Europe and Asia and the causative agent of tick-borne encephalitis (TBE). Annually more than 10,000 TBE cases are reported despite having vaccines available. In Europe, the vaccines FSME-IMMUN® and Encepur® based on formaldehyde-inactivated whole viruses are licensed. However, demanding vaccination schedules contribute to sub-optimal vaccination uptake and breakthrough infections have been reported repeatedly. Due to its immunogenic properties as well as its role in viral replication and disease pathogenesis, the non-structural protein 1 (NS1) of flaviviruses has become of interest for non-virion based flavivirus vaccine candidates in recent years.

**Methods:**

Therefore, immunogenicity and protective efficacy of TBEV NS1 expressed by neuraminidase (NA)-deficient Influenza A virus (IAV) or Modified Vaccinia virus Ankara (MVA) vectors were investigated in this study.

**Results:**

With these recombinant viral vectors TBEV NS1-specific antibody and T cell responses were induced. Upon heterologous prime/boost regimens partial protection against lethal TBEV challenge infection was afforded in mice.

**Discussion:**

This supports the inclusion of NS1 as a vaccine component in next generation TBEV vaccines.

## 1 Introduction

The neurotropic tick-borne encephalitis virus (TBEV) belongs to the genus Flavivirus of the *Flaviviridae* family and is one of the most important tick-transmitted viruses in Europe and Asia. Since 2000, more than 50,000 confirmed tick-borne encephalitis (TBE) cases have been reported in Europe and the number of countries reporting cases is increasing [reviewed in (1)]. Its positive-sensed single-stranded RNA genome encodes for a polyprotein that is cleaved co- and post-translationally by viral and cellular proteases into three structural proteins (capsid C, envelope E, precursor membrane/membrane prM/M) and seven non-structural (NS) proteins (NS1, NS2A, NS2B, NS3, NS4A, NS4B, and NS5). Three main TBEV subtypes are distinguished, the Far-Eastern, the European and the Siberian subtype, which differ in geographical spread and virulence. More recently, novel Baikalian and the Himalayan subtypes have been identified (2–4). TBEV is mainly transmitted by *Ixodes* spp. ticks (5) and causes asymptomatic infections in humans in 70-98% of cases depending on viral (e.g. dose, virulence of TBEV strain) and host factors (e.g. age) [reviewed in (6)]. However, patients can also present with a wide range of clinical signs, from mild febrile illness to severe neurological disease including meningitis, encephalitis and myelitis, summarized as TBE. Up to 50% of patients with TBE can suffer from long-term sequelae [reviewed in (7)]. Specific antiviral treatment of TBE is not available in Europe [reviewed in (6)] and therefore, vaccination is the most important protective measure. Worldwide, six inactivated TBEV vaccines have been licensed. In Europe, FSME-IMMUN® (Pfizer) and Encepur® (Bavarian Nordic), both based on European TBEV strains, are being used. For primary vaccination, three vaccine doses are required with the need of booster vaccinations every 3-5 years depending on the age of the vaccinee. Although these vaccines are considered safe and effective with high seroconversion rates (8), vaccine breakthrough infections have been reported to occur frequently (9–14). Of interest, TBE in patients with vaccination breakthrough has been described to be more severe than after infection in unvaccinated patients (9, 12), reviewed in (6)]. TBEV vaccination aims primarily at the induction of virus-neutralizing antibodies to the E protein. In addition, it has been shown that the currently used vaccines also induce virus-specific CD4^+^ T cell responses [reviewed in (15)]. Some studies indicate that the use of European licensed vaccines also induce NS1-specific antibodies, however, this is still matter of debate (16–18). NS1 plays an important role in the TBEV replication cycle and may contribute to the pathogenesis of TBEV infections as was described for other flaviviruses [reviewed in (19)]. NS1 is involved in viral replication and virus assembly intracellularly, but it is also found on the cell surface and can be secreted into extracellular space in its oligomeric form (20). Although the mechanisms of protection are not fully understood, it was shown previously that immunization with TBEV NS1 is able to partly protect mice against challenge infection (21–27). For the induction of TBEV NS1-specific immunity, synthetic peptides (25, 26) and recombinant viral vectors based on adenoviruses (21, 22, 27) and vaccinia viruses (VACV) (23, 24) have been used. The use of viral vectors offers the advantage that they can induce both humoral and cell-mediated immunity, although the latter has not been studied in great detail.

The use of NS1 as vaccine antigen may offer some advantages over the use of the E protein. NS1 vaccination will not result in the induction of virion-reactive antibodies and consequently, the risk of antibody-dependent enhancement (ADE) of infection is reduced (28). Although clear evidence for ADE of TBEV infection *in vivo* is lacking, ADE has been shown *in vitro* (29–32).

In the present study, we constructed and characterized Influenza A virus (IAV)- and Modified Vaccinia virus Ankara (MVA)-based vectors expressing the TBEV NS1 protein (IAV-NS1 and MVA-NS1) and tested their immunogenicity and protective efficacy in a mouse model. Recombinant IAVs, member of the *Orthomyxoviridae* family, have been used for the development of vaccine candidates against a variety of different viruses, and immunogenicity was demonstrated in pre-clinical studies [reviewed in (33)]. MVA, a member of the *Poxviridae* family, has a longstanding record as a safe and effective viral vaccine vector with extensive use in clinical trials [reviewed in (34)]. To investigate whether TBEV NS1-specific antibody and T cell responses can be improved, we also evaluated heterologous prime/boost vaccination regimens with IAV and MVA as viral vectors. Since the order of prime/boost administration can be important [reviewed in (35)], prime immunization with MVA-NS1 followed by boost immunization with IAV-NS1 and *vice versa* was tested. Heterologous prime/boost regimens with MVA-NS1 and IAV-NS1 proved to be highly immunogenic, induced NS1-specific antibodies, CD4^+^ and CD8^+^ T cell responses and afforded partial protection against a lethal TBEV challenge in mice.

## 2 Materials and methods

### 2.1 Cell culture

All cell lines were cultivated at 37°C in 5% CO_2_. Primary chicken embryo fibroblast (CEF) cells were generated from 10-11 days old chicken embryos (specific pathogen-free eggs purchased from VALO BioMedia GmbH) and cultured in Minimum Essential Medium Eagle (Sigma-Aldrich) with 10% fetal bovine serum (FBS), 100 IU/ml penicillin and 100 µg/ml streptomycin (Pen/Strep, Sigma-Aldrich), and 1% MEM non-essential amino acid solution (NEAA, Sigma-Aldrich). HEK293T and MDCK cells were propagated in Dulbecco’s modified Eagle medium (DMEM, Gibco™) supplemented with 10% FBS, Pen/Strep and 2 mM GlutaMAX™ (Gibco™). HeLa cells were cultivated in DMEM with 10% FBS, Pen/Strep, 2 mM GlutaMAX™ and 1% MEM NEAA solution. A549 cells were maintained in F-12 Nut Mix (1X) + GlutaMAX-I (Gibco™) supplemented with 10% FBS, Pen/Strep, 2 mM GlutaMAX™ and 20 mM HEPES. For IAV infection, 10% FBS was replaced by 0.1% bovine serum albumin and 1 µg/ml Trypsin-TPCK (Sigma-Aldrich) was added freshly to the media. For MVA and TBEV infection media, 10% FBS was replaced by 2% FBS. Cell lines were tested negative for mycoplasma before use (MycoStrip™–Mycoplasma detection Kit, InvivoGen).

### 2.2 Viruses

TBEV strain Neudoerfl (European subtype) was provided by the Department of Microbiology of the German Armed Forces, Munich, Germany. The reverse genetics pHW2000 plasmids containing the individual gene segments of A/Puerto Rico/8/1934 (H1N1) (PR8) were provided by Richard Webby and Robert Webster, St. Jude Children’s Research Hospital, Memphis, TN, USA. Plasmids encoding for TBEV Neudoerfl NS1 (based on UniProtKB: P14336) and the SARS-CoV-2 receptor binding domain (RBD, aa319-550, based on Wuhan-Hu-1 GenBank: MN908947.3) including a C-terminal stop codon and EcoRI and SpeI restriction sites at the 5’- and 3’-end, respectively, were synthesized (GenScript Biotech Corp) and cloned in frame into the previous used pHW2000 neuraminidase (NA) plasmid encoding a fusion of the N-terminal region of PR8 NA with enhanced green fluorescent protein (eGFP) (36). Thereby, eGFP was replaced by TBEV NS1 or the SARS-CoV-2 RBD as non-TBEV insert. By using reverse genetics based on the 8 plasmid system (37), recombinant PR8 (rPR8), IAV-NS1 and IAV-RBD (vector control virus) were rescued. For this, HEK293T cells were transfected with plasmids encoding the respective chimeric or wildtype NA and the remaining PR8 gene segments by using TransIT®-LT1 (Mirus Bio LLC). Infection medium was supplemented with exogenous *Vibrio cholerae* neuraminidase (eNA, 1:60,000, Sigma). After 48 h, HEK293T supernatant was transferred to MDCK cells for virus amplification. To generate IAV stocks, MDCK cells were infected with multiplicity of infection (MOI) 0.01 of the respective virus in presence of eNA. 48 hours post infection (hpi), supernatant was harvested and clarified by low-speed centrifugation. Stocks for animal experiments were subsequently concentrated 120x by ultracentrifugation through a 25% sucrose cushion (28,000 rpm, 2 h, 4°C). Virus pellets were resuspended in 1x PBS (Gibco™). Viral titers were determined by plaque assay as described before with avicel overlay (38).

**Figure 1:**
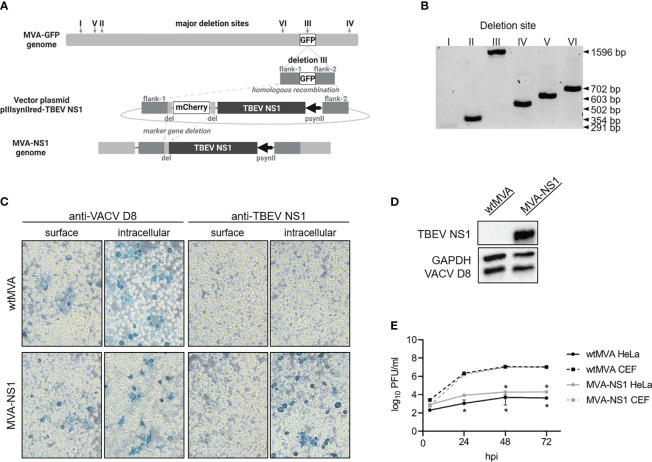
*In vitro* characterization of MVA-NS1. **(A)** Homologous and intragenomic homologous (marker gene deletion) recombinations lead to production of MVA-NS1 with TBEV NS1 expression under transcriptional control of VACV late promotor psynII. **(B)** PCR products specific for the six major deletion sites inside the MVA genome performed on MVA-NS1 (1% agarose TBE gel) (I: 291 bp, II: 354 bp, III: 447 bp, IV: 502 bp, V: 603 bp, VI: 702 bp). Integration of the NS1 gene in deletion site III was confirmed (III: 1596 bp). **(C)** Immunostaining of wtMVA or MVA-NS1 infected HeLa cells (MOI 1). 24 hpi, cells were fixed with 4% PFA, for intracellular staining permeabilized with TritonX®-100 and immunostained against VACV D8 and TBEV NS1 (20x magnification). **(D)** Western blot analysis of whole cell lysate from HeLa cells infected with wtMVA or MVA-NS1 for 24 h (MOI 5). Blots were stained against TBEV NS1. For control, antibodies against VACV D8 and GAPDH were included. **(E)** Growth curves of wtMVA (black) and MVA-NS1 (gray) on primary CEF cells (dotted line) and HeLa cells (solid line) (MOI 0.05). Mann-Whitney test was used for statistical comparison between CEF and HeLa cells (* p<0.05).

Non-recombinant MVA F6 (wtMVA) and MVA-GFP (expression of GFP under transcriptional control of VACV promotor P11 in deletion site III) were obtained from the Institute for Infectious Diseases and Zoonoses, Ludwig Maximilian University Munich, Munich, Germany. Plasmid encoding for the Kozak sequence followed by the TBEV E gene signal peptide and entire TBEV NS1 were synthesized (based on TBEV Neudoerfl, UniProtKB: P14336; GenScript Biotech Corp) and cloned into MVA transfer plasmid pIIIsynIIred under transcriptional control of VACV late promotor psynII (39). pIIIsynIIred contains mCherry as marker gene which is flanked by short repetitive regions. MVA-NS1 was produced as described previously (39) (**Figure 1A**). Virus stocks were propagated on primary CEF cells and virus was concentrated by ultracentrifugation at 38,400 rcf through 36% sucrose cushion. Virus pellets were resuspended in tris-buffered saline (120 mM NaCl/10 mM Tris-HCl, pH 7.4). MVA-specific immune peroxidase staining after slightly modified standard protocol (39) including overlay consisting of 2.5% Avicel in 2X MEM, 2% FBS, 1% Pen/Strep and 1% MEM NEAA solution was performed to determine viral titers.

All viruses were stored at -80°C and tested negative for mycoplasma before use (MycoStrip™–Mycoplasma detection Kit, InvivoGen).

### 2.3 *In vitro* characterization of vector constructs

#### 2.3.1 Integration of NS1 and sequence analysis

IAV RNA was isolated using the QIAmp® Viral RNA Mini Kit (Qiagen) following manufacturer’s instructions and reverse transcribed into cDNA using SuperScript III reverse transcriptase (Invitrogen) with the Uni12 primer (5’-AGCAAAAGCAGG-3’) (40). Chimeric NA cDNA regions were amplified using AmpliTag Gold DNA polymerase (Applied Biosystems) with primers NA int for (5´-ATCTGTCTGGTAGTCGGA-3´) and NA int rev (5´-GGCCAAGACCAATCTACA-3´). For amplification of the hemagglutinin (HA) gene segment, primers HA for (5’-AGCAAAAGCAGGGG-3’) and HA rev (5’-AGTAGAAACAAGGGTGTTTT-3’) were used. PCR products were separated on 0.8% agarose TBE gel and sequence identity of NA and HA was confirmed by sequencing (Microsynth Seqlab).

NS1 gene sequence integration into deletion site III of the MVA genome was verified by PCRs specific for the six major deletion sites of MVA as described previously (39). Purified PCR products were separated on 1% agarose TBE gel and analyzed with imaging system (ChemiDoc, ImageLab v6.0.1, Bio-Rad Laboratories, Inc.). For sequencing of NS1, deletion site III-specific PCR was performed (39) and purified PCR product was sequenced (Microsynth Seqlab). For PCRs, GoTaq® DNA polymerase (Promega) and for DNA purification, GeneJET Gel Extraction Kit (Thermo Scientific™) were used.

#### 2.3.2 Western blot and immunostaining

To show expression of TBEV NS1 by the IAV vector system, MDCK cells were infected with MOI 0.01 of the respective IAV in the absence of eNA. For MVA vector system, HeLa cells were infected with MOI 5 of the respective MVA. For Western blot analysis, cells were lysed 24 hpi using RIPA buffer with 1% Halt™ Protease-Inhibitor-Cocktail (100x) (Thermo Fisher Scientific). Lysates were separated on 10% SDS-PAGE gels (Bio-Rad Laboratories, Inc.) and blotted on Cytiva Amersham™ Hybond™ P 0.45μm PVDF Membrane (VWR). Proteins were stained by using the mouse anti-TBEV NS1 monoclonal antibody (mAb) (M838, The Native Antigen Company), rabbit anti-GAPDH (D16H11) XP® mAb (Cell Signaling Technology®), mouse anti-influenza NP mAb (ATCC, clone HB65) or polyclonal rabbit anti-Cell Surface-Binding Protein (D8L) antibody (Biozol), respectively. After incubation with goat anti-rabbit IgG (H+L) HRP (Invitrogen) or goat anti-mouse IgG (H+L) HRP (Invitrogen) as secondary antibodies, blots were developed using SuperSignal™ West Pico PLUS Chemiluminescent Substrate (Thermo Scientific™) and ChemiDoc Imaging System (Bio-Rad Laboratories, Inc.). For immunostaining, infected cells (IAV: MDCK cells, MOI 0.01; MVA: HeLa cells, MOI 1) were fixed 24 hpi with 4% ice-cold paraformaldehyde (PFA, Roth). For intracellular staining, cells were permeabilized using 0.5% Triton X®-100 (Roth). Viral proteins were detected by using the above mentioned antibodies. For visualization, stained cells were incubated with HRP-substrate TrueBlue™ Peroxidase Substrate (SeraCare) supplemented with 1:1000 H_2_O_2_ (Roth) and imaged with Leica DM IL LED microscope.

#### 2.3.3 Growth kinetics

MDCK cells were infected in the presence or absence of eNA with MOI 0.001 of the respective IAV. For MVA, primary CEF and HeLa cells were infected with MOI 0.05. Supernatant was taken 2/4, 24, 48 and 72 hpi and viral titers were determined by plaque assay as described above.

### 2.4 Animal experiments

#### 2.4.1 Ethical statement

All animal experiments were conducted at the University of Veterinary Medicine Hannover, Foundation, Hannover, Germany in strict compliance with European guidelines (EU directive on animal testing 2010/63/EU) and German Animal Welfare Law. The study protocol was approved by the Lower Saxony State Office for Consumer Protection and Food Safety (LAVES, Approval No. 33.8-42502-04-20/3437).

#### 2.4.2 Mice

Female C57BL/6JOlaHsd (BL6) mice were purchased from the commercial breeder Envigo RMS. Depending on the required biosafety level, mice were housed in individually ventilated cages type Sealsafe Plus GM500 or IsoCage N Biocontainment System (Tecniplast), respectively. Sterilized food pellets and water were provided *ad libitum*. All experiments started after at least one week of habituation and acclimatization of mice. Treatment of mice was done under isoflurane anesthesia.

#### 2.4.3 Immunogenicity study

Six to eight weeks old BL6 mice (n=4/group) were vaccinated twice in a 4-week interval with 10^7^ plaque-forming units (PFU) of the IAV [subcutaneous, dorsally in the neck region (s.c.) as described previously (41)] or MVA [intramuscular, left hind limb (i.m.)] vector constructs. 170 µl of FSME-IMMUN® 0.5 mL (Pfizer, lot number EM2898) was administered as positive control (0.816 µg/mouse; s.c.). 100 µl PBS (s.c.) was administered as negative (mock) control. To minimize the number of experimental animals and to comply with the 3R principle (replacement, reduction and refinement), data of wtMVA-vaccinated mice (empty vector control group) were shared with an experiment performed in parallel under identical experimental conditions (same approval number). This was deemed justified because many studies failed to demonstrate any effect of MVA vector control induced immunity on immune responses to the pathogen of interest and protective efficacy (e.g (42–46). Mice were bled on day 0 and 28 before the first and second vaccination, respectively, by puncturing *Vena facialis* and on day 56 by retrobulbar sinus puncture. Blood was collected in MiniCollect® CAT Serum Sep Clot Activator tubes (Greiner Bio-One GmbH), incubated 30 min at room temperature (RT) and centrifuged at 3000 x*g* for 10 min to collect serum. After final blood drawing, mice were euthanized and spleens from individual mice were collected for the generation of single-cell suspensions through cell strainers followed by erythrocyte lysis using ACK Lysing buffer (Gibco™). Splenocytes were resuspended in RPMI 1640 (1X) with 10% FBS, Pen/Strep and 5 mM ß-mercaptoethanol (R10F) and directly used for ELISpot and FACS assays.

#### 2.4.4 TBEV challenge infection

All challenge infection experiments were done under biosafety level 3**. Mice (n=12/group) were vaccinated as described above. On day 56 post prime immunization, blood was taken at *Vena facialis* and mice were infected with 5.4x10^3^ TCID_50_ TBEV strain Neudoerfl (100 µl, s.c.). Upon challenge infection, mice were scored daily based on the clinical score sheet including the categories outer appearance, activity, movement, body weight and neurological signs. Half of the mice (n=6/group) were euthanized 8 days post infection (dpi) for determination of viral loads, remaining mice (n=6/group) were taken out of the experiment according to humane endpoint (HEP) or study endpoint (SEP, 16 dpi). On day of sacrifice, mice were bled by retrobulbar sinus puncture and euthanized. Organs (left brain hemisphere, cervical part of spinal cord, spleen, rice-corn sized part of ileum and colon) were collected in 1 ml PBS, homogenized with a stainless steel bead using the TissueLyser II (Qiagen) with 30 Hz for 1 min and stored at -80°C. The right brain hemisphere and remaining gastrointestinal tract were fixed in ROTI®Histofix 4% (Roth, for at least 48 h) for histopathological analysis.

### 2.5 Serology

#### 2.5.1 Enzyme-linked immunosorbent assay

To detect TBEV NS1-specific IgG antibodies, Mouse Anti-Tick Borne Encephalitis Virus Non-Structural Protein 1 IgG Elisa Kit (Alpha Diagnostic International) was used according to manufacturer’s instructions. Serum was heat-inactivated before use (30 min, 56°C). Concentration of specific anti-NS1 antibodies was measured in arbitrary units (U/ml).

#### 2.5.2 Luciferase immunoprecipitation systems assay

Luciferase Immunoprecipitation System (LIPS) assay for TBEV NS1 was performed as described previously (47) with 1:100 diluted, heat-inactivated mouse sera (30 min, 56°C). LIPS plasmids were kindly provided by Imke Steffen (Institute for Biochemistry and Research Center for Emerging Infections and Zoonoses, University of Veterinary Medicine Hannover, Foundation, Hannover, Germany). Luminescence was measured using the microplate reader infinite 200Pro (Tecan) and Tecan i-control software (version 2.0.10.0, Tecan). Average of triplicate measurements was determined and expressed in relative light units (RLU). RLU values higher than the average of negative samples plus five times standard deviation are considered positive.

#### 2.5.3 Virus neutralization assay

To test mouse sera for TBEV-neutralizing antibodies, heat-inactivated sera (30 min, 56°C) were two-fold serially diluted in A549 infection medium, starting with a 1:10 dilution. 100 TCID_50_/well TBEV Neudoerfl was added and incubated for 1 h at 37°C. Subsequently, virus-serum mix was transferred in triplicates to 80% confluent A549 cells which were incubated for 5-6 days at 37°C, 5% CO_2_. Reduction of the cytopathic effect (CPE) by 100% compared to the negative serum control was considered as virus neutralization. Virus neutralizing titers (VNT_100_) were defined microscopically as the reciprocal of the highest serum dilution still resulting in complete inhibition of CPE.

#### 2.5.4 Hemagglutination inhibition assay

To demonstrate IAV-specific antibodies, heat-inactivated (30 min, 56°C) mouse sera were tested in hemagglutination inhibition (HI) assay as described before (48). In short, serum was pre-incubated for 16 h at 37°C with *Vibrio cholerae* filtrate (generously provided by Ron Fouchier, Erasmus Medical Center, Rotterdam, Netherlands) and subsequently heat-inactivated at 56°C for 1 h. HI assay was then performed after standard protocol with 4 hemagglutination units of rPR8 and 1% chicken erythrocytes.

### 2.6 Splenocytes

#### 2.6.1 Restimulation of splenocytes

TBEV-specific peptide pools based on TBEV Neudoerfl NS1 (UniProtKB: P14336) were used for the *ex vivo* restimulation of splenocytes. Lyophilized 15-mer peptides with 11 amino acid overlaps (≥ 75% purity, GenScript Biotech Corp) were dissolved in DMSO and two pools were generated (NS1_1-183_: 43 peptides; NS1_173-352_: 42 peptides). For *ex vivo* restimulation, mouse splenocytes were incubated overnight at 37°C/5% CO_2_ with a final peptide concentration of 1 µg/ml, 30 ng/ml Phorbol 12-myrisate 13-acetate (PMA; Cayman Chemical) and 0.5 µg/ml Ionomycin (Cayman Chemical) (positive control), MOI 1 of live IAV or MOI 3 of live MVA, and DMSO/R10F (negative control), respectively.

#### 2.6.2 IFN-γ ELISpot

IFN-γ ELISpot assays were performed with Mouse IFN-γ ELISpot^PLUS^ kit (ALP) (Mabtech) according to manufacturer’s standard protocol. Mouse splenocytes were restimulated as described above on pre-coated 96-well ELISpot plates in the presence of antigen/control for 20 h at 37°C, 5% CO_2_. After staining, spots were scanned and counted by using the ImmunoSpot® S6 Ultimate Reader and the ImmunoSpot® software (Version 7.0.20.1, Immunospot, CTL). Triplicates were averaged and data expressed as spot-forming cells (SFC) per 10^6^ splenocytes after background subtraction (DMSO/R10F stimulation).

#### 2.6.3 Flow cytometry

Mouse splenocytes (1x10^6^ cells/well) were restimulated as described above. For the final 4 h of restimulation, Brefeldin A (10 µg/ml, Sigma-Aldrich) was added to the medium. Cells were stained with LIVE/DEAD™ Fixable Near-IR Dead Cell Stain Kit for 633 or 635 nm excitation (Invitrogen™) for 20 min in the dark followed by Fc receptor blocking with anti-Mouse CD16-CD32 (Clone: 93) for 15 min at RT in the dark. Surface staining was performed for 20 min at 4°C in the dark. Cells were then permeabilized and fixed with BD Cytofix/Cytoperm™ (BD Biosciences) for 20 min at 4°C in the dark, intracellular staining was performed for 30 min at 4°C in the dark. After resuspension in PBS, cells were acquired by BD LSR Fortessa X-20 (BD Biosciences) using BD FACSDiva (version 9.0, BD Biosciences). Used antibodies are listed in **Table 1** and were used at 1:200 dilution.

**Table 1:** Antibody panel used in flow cytometry experiments.

Antibody	Fluorochrome	Vendor	Clone
CD3e	FITC	eBioSciences™	145-2C11
CD4	PE	eBioSciences™	RM4-5
CD8a	PerCP-Cyanine5.5	eBioSciences™	53-6.7
IFN-γ	APC	eBioSciences™	XMG1.2
Granzyme B	BV421	BioLegend®	QA18A28
CD69	Alexa Fluor® 700	BD Biosciences	H1.2F3

Antibodies used for surface and intracellular staining for flow cytometric analysis with their respective details are listed.

### 2.7 Determination of live virus in serum and organs by tissue culture infectious dose 50%

Tissue samples were thawed and tissue debris was removed by centrifugation (3000 x*g*, 10 min). 80% confluent A549 cells were inoculated in quadruplicates with 10-fold serial dilutions of sera or organ samples prepared in A549 infection medium. After 5-6 days at 37°C, 5% CO_2_, cells were screened for the presence/absence of CPE. TCID_50_ values for individual samples were determined using the Reed and Muench method (49) and calculated to 1 g tissue (TCID_50_/g tissue) or 1 ml (TCID_50_/ml). Detection limit for each organ was defined by dividing the lowest dilution (10^1^) by the respective averaged organ weight.

### 2.8 RNA isolation and real time reverse transcriptase quantitative PCR

Total RNA was extracted from sera and clarified organ homogenate supernatants using the QIAmp® Viral RNA Mini Kit (Qiagen) following manufacturer’s protocol. Real time reverse transcriptase quantitative PCR (RT-qPCR) was performed with Luna® Universal One-Step RT-qPCR Kit (New England BioLabs® GmbH) based on the modified protocol by Schwaiger and Cassinotti (50) including a TBEV Neudoerfl RNA standard kindly provided by Stefanie Becker (Institute for Parasitology and Research Center for Emerging Infections and Zoonoses at University of Veterinary Medicine Hannover, Foundation, Hannover, Germany). Real time RT-qPCR was run in duplicates using AriaMx Real-time PCR System (Agilent Technologies) with Agilent Aria software (version 1.5, Agilent Technologies). Absolute copy numbers were calculated based on the standard curve and expressed as TBEV copies/ml or g tissue.

### 2.9 Histology

Two longitudinal sections of the right brain hemisphere and representative sections of duodenum, jejunum, ileum, caecum, colon and rectum were embedded in paraffin wax followed by cutting 2-3 µm thick sections using a microtome. Sections were stained with hematoxylin and eosin (H&E) and analyzed as described previously (51). Briefly, three brain regions (olfactory bulb, cerebral cortex, hippocampus) were selected for histopathological analysis and evaluated with respect to microscopic lesions including perivascular as well as vascular inflammation, vascular lesions including perivascular edema, hemorrhage and fibrinoid necrosis, microgliosis characterized by hyperplasia and/or hypertrophy of microglia/macrophages as well as cellular necrosis characterized by karyorrhexis, karyolysis, pyknosis and triangularly shaped, hypereosinophilic and shrunken neurons. To assess histological changes in the intestine of mice, submucosal and myenteric plexus were investigated with respect to microscopic lesions including necrosis of ganglion neurons characterized by karyorrhexis, karyolysis and pyknosis, hypereosinophilia and shrinkage of neurons as well as presence of inflammatory cell infiltrates/hypercellularity in plexus.

### 2.10 Immunohistochemistry

For immunohistochemistry (IHC), the avidin-biotin-peroxidase (ABC) complex method was applied as previously published (52). For immunohistochemical detection of TBEV antigen, a mouse anti-TBEV E mAb (clone 19/1493, diluted 1:2000, kindly provided by Matthias Niedrig) was used. Sections of brain and intestine were examined with respect to the presence of TBEV-antigen positive cells as described previously (51).

### 2.11 Statistics

GraphPad Prism software (version 9.0.0, GraphPad Software Inc.) was used for statistical analysis. Statistical test used for analyses are stated in the respective figure legends. A p value <0.05 was considered as significant.

## 3 Results

### 3.1 TBEV NS1 is expressed by the highly attenuated IAV- and MVA-vectors

**Figure 2:**
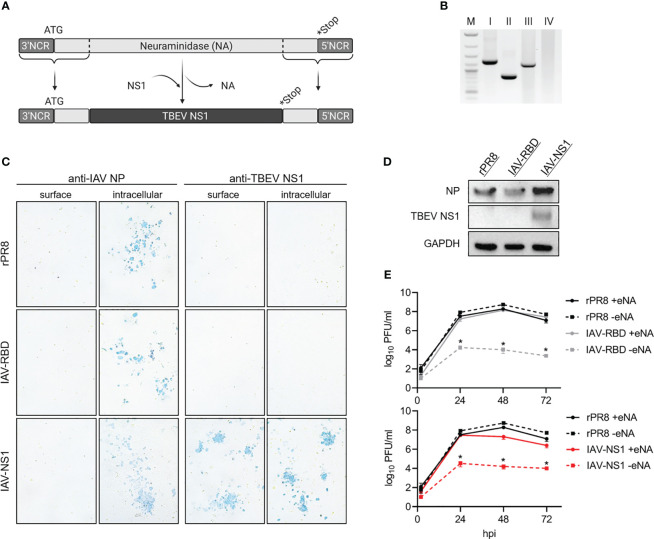
*In vitro* characterization of IAV-NS1. **(A)** Schematic representation of the NA-TBEV NS1 gene segment, NCR = non-coding region, ATG = start codon, *Stop = stop codon. **(B)** PCR products specific for IAV NA performed on I = rPR8, II = IAV-RBD, III = IAV-NS1 and IV = negative control (1% agarose TBE gel, M = 1 kb ladder). **(C)** Immunostaining of rPR8, IAV-RBD or IAV-NS1 infected MDCK cells (MOI 0.01). 24 hpi, cells were fixed with 4% PFA, for intracellular staining permeabilized with TritonX®-100 and immunostained against IAV NP or TBEV NS1 (10x magnification). **(D)** Western blot analysis of whole cell lysate from MDCK cells infected with rPR8, IAV-RBD or IAV-NS1 for 24 h (MOI 0.01). Blots were stained against TBEV NS1. For control, antibodies against IAV nucleoprotein (NP) and GAPDH were included. **(E)** Growth curves for rPR8 (black), IAV-RBD (gray) and IAV-NS1 (red) in the presence (solid line) or absence (dotted line) of eNA on MDCK cells (MOI 0.001). Mann-Whitney test was used for statistical comparison between rPR8 –eNA and IAV-RBD –eNA or IAV-NS1 –eNA, respectively (* p<0.05).

Using reverse genetics (37), the PR8-based virus IAV-NS1 harboring a chimeric NA gene segment was rescued. Most of the open reading frame of the IAV NA gene was replaced by the NS1 gene of the TBEV strain Neudoerfl terminated by a stop codon. Essential IAV packaging signals located in the 3’ and 5’ ends were maintained (53). This way, IAV-NS1 expresses TBEV NS1 as a fusion protein with the N-terminal region of the NA of IAV PR8 (**Figure 2A**). Accordingly, the vector control virus IAV-RBD expressing a non-TBEV gene was designed and rescued. Correct insertion into NA was confirmed by RT-PCR specific for the NA gene (**Figure 2B**). Nucleotide sequences of the HA and chimeric NA genes and absence of mutations were confirmed by Sanger sequencing (data not shown). MVA served as a second viral vector system delivering TBEV NS1 (**Figure 1A**). Correct integration of the NS1 gene in deletion site III of the MVA genome was verified by PCRs specific for the six major MVA deletion sites (**Figure 1B**). Additionally, nucleotide sequencing confirmed complete insertion of the correct NS1 sequence (data not shown).

Expression of TBEV NS1 was shown by Western blot analysis of lysates of MDCK cells infected with IAV-NS1 or HeLa cells infected with MVA-NS1 (**Figures 1D**, **2D**). Immunostaining of respective cells infected with IAV-NS1 or MVA-NS1 confirmed the intracellular expression of TBEV NS1 (**Figures 1C**, **2C**). In addition, cell surface expression of NS1 in non-permeabilized cells was shown for IAV-NS1 (**Figure 2C**).

Insertion of foreign genes should not affect the attenuated phenotype of vector-based vaccine constructs. Therefore, growth characteristics of the vector controls, IAV-NS1 and MVA-NS1 were analyzed by performing multi-step growth kinetics (**Figures 1E**, **2E**). The vector control IAV-RBD and IAV-NS1 displayed a highly attenuated growth phenotype compared to the recombinant wildtype virus rPR8 in the absence of eNA. However, addition of eNA to the cell culture medium restored the *in vitro* replicative capacity of IAV-RBD and IAV-NS1. Viral titers peaked at 48 hpi and declined thereafter. rPR8 replicated independently of eNA to high titers (**Figure 2E**). Similarly, wtMVA and MVA-NS1 replicated well in permissive primary CEF cells up to titers of 10^7^ PFU/ml. However, in non-permissive human HeLa cells, replication-deficiency for wtMVA and MVA-NS1 was confirmed (**Figure 1E**).

Thus, two attenuated vector-constructs based on IAV and MVA were constructed that drive the expression of the TBEV NS1 gene. Subsequently, we tested the tolerability and immunogenicity in mice.

### 3.2 IAV-NS1 and MVA-NS1 are immunogenic in mice

To test the immunogenicity of the respective NS1 vector constructs, mice were vaccinated twice at a 4-weeks interval with 10^7^ PFU of IAV-NS1 or MVA-NS1. For heterologous prime/boost vaccination regimens, we immunized mice with MVA-NS1 followed by an IAV-NS1 booster immunization (MVA-NS1/IAV-NS1) and *vice versa* (IAV-NS1/MVA-NS1). As controls, mice were vaccinated twice with 0.816 µg FSME-IMMUN®, PBS or vector controls (IAV-RBD, wtMVA).

All vaccine preparations and regimens were well tolerated and all mice continued to gain weight over the course of immunization without displaying any clinical signs (**Supplementary Figure S1**). Eight weeks after the first immunization, serum samples and splenocytes were collected to analyze TBEV-specific immune responses.

**Figure 3:**
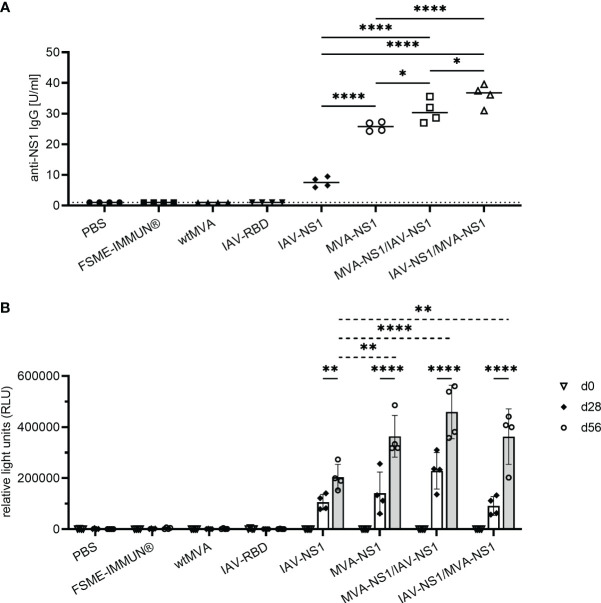
Antibody response against TBEV NS1. **(A)** Quantitative measurement of TBEV NS1-specific IgG by NS1 ELISA of sera samples collected 56 days post prime immunization. Results are reported as arbitrary units per ml (U/ml). Cut-off values were calculated according to the manufacturer’s instructions (1 U/ml). One-way ANOVA with Tukey’s multiple-comparison test was used for statistical analysis. Significances are shown for all NS1-specific vaccine groups (* p<0.05, **** p<0.0001). **(B)** Semi-quantitative measurement of TBEV NS1-specific antibodies by LIPS assay with mouse sera from day 0, 28 and 56 post prime immunization in relative light units (RLU). Mean values with SD are shown from 2-5 independent experiments (n=4 mice/group). Two-way ANOVA with Tukey’s multiple-comparison test was used for statistical analysis. Significances are shown for RLU values on d28 vs. d56 (solid line) and for all NS1-specific vaccine groups compared at d56 (dotted line) (** p<0.01, **** p<0.0001).

None of the mock or vector control-vaccinated mice developed TBEV-specific antibodies as measured by NS1-specific ELISA, LIPS assay and VN assay (**Figure 3**, **Supplementary Figure S2**). In contrast, all mice vaccinated with IAV-NS1 or MVA-NS1 developed TBEV NS1-specific antibodies after a single immunization. These antibody levels were boosted by a second immunization (**Figure 3B**). Two doses with MVA-NS1 induced higher antibody levels than two doses of IAV-NS1. With the heterologous prime/boost regimens, significantly higher TBEV NS1 antibody levels were achieved (**Figures 3A, B**). Vaccination with FSME-IMMUN® did not induce TBEV NS1-specific antibodies (**Figure 3**), but resulted in the induction of high VN antibody titers (**Supplementary Figure S2**). The use of NS1-based vaccine preparations did not result in the induction of VN antibodies (**Supplementary Figure S2**).

Vaccination with the IAV-vector induced high titers of PR8-specific antibodies as measured by HI assay. Mice receiving IAV-NS1 prime and MVA-NS1 boost developed slightly lower IAV-specific HI titers compared to the other groups immunized with IAV-based vector constructs. Mice of all other treatment groups were negative in the HI assay (**Supplementary Figure S3**).

**Figure 4:**
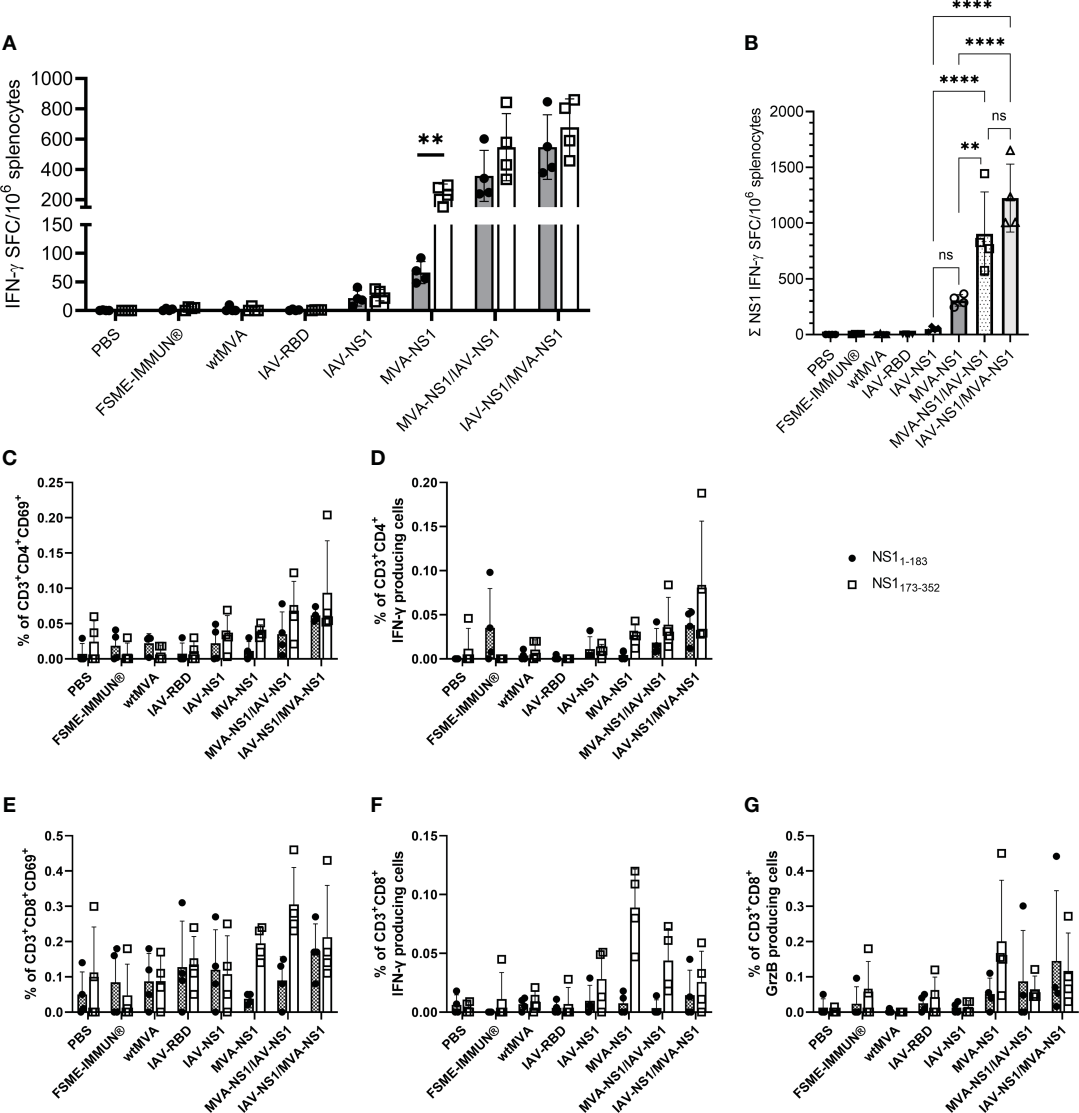
T cell response against TBEV NS1. **(A)** For IFN-γ ELISpot, mouse splenocytes were restimulated overnight with TBEV NS1 peptide pools NS1_1-183_ and NS1_173-352_. Mean values with SD are shown as IFN-γ spot-forming cells (SFC) per one million splenocytes after background subtraction. For statistical analysis, unpaired t-test was used (**p<0.01). **(B)** Calculated IFN-γ SFC per million splenocytes for total NS1 (NS1_1-183_ + NS1_173-352_). One-way ANOVA with Tukey’s multiple-comparison test was used for statistical analysis. Significances are only shown for NS1-vaccine groups (ns = not significant, ** p<0.01, **** p<0.0001). **(C–G)** Flow cytometric analysis of mouse splenocytes. Frequency of CD3^+^ subpopulations gated on CD4^+^ **(C, D)** and CD8^+^ **(E–G)** T cells positive for CD69, IFN-γ and Granzyme B (GrzB) upon restimulation with NS1_1-183_ (black circle) or NS1_173-352_ (unfilled square) (n = 4). Bars represent mean with SD. Data is shown after background subtraction.

NS1-specific T cell responses were analyzed by IFN-γ ELISpot assay using splenocytes restimulated with peptide pools of TBEV NS1 (NS1_1-183_ and NS1_173-352_). IFN-γ SFC were detectable in all mice that received IAV-NS1 and/or MVA-NS1 (**Figure 4A**). Splenocytes from all NS1-vaccinated mice responded to both peptide pools, however, the response to NS1_173-352_ peptide pool was in general higher than the response to NS1_1-183_. Mice that received heterologous prime/boost regimens displayed significantly higher combined numbers of IFN-γ positive spots than the mice that received IAV-NS1 or MVA-NS1 only (**Figure 4B**). In FSME-IMMUN®-vaccinated and control mice, NS1-specific IFN-γ SFC were not detected. Further flow cytometric analysis revealed that both antigen-specific CD4^+^ and CD8^+^ T cells were the source of IFN-γ. In agreement with the ELISpot data, frequencies of NS1_173-352_-specific IFN-γ^+^ cells were higher than those specific for NS1_1-183_ (**Figures 4D, F**). Moreover, CD8^+^ T cells positive for Granzyme B were detected in mice that received two doses of MVA-NS1 or heterologous prime/boost regimens (**Figure 4G**). The induction of NS1-specific CD4^+^ and CD8^+^ T cells was further confirmed by the detection of T cells expressing the early activation marker CD69 (**Figures 4C, E**). The respective viral vectors also induced vector-specific CD4^+^IFN-γ^+^, CD8^+^IFN-γ^+^ and CD8^+^Granzyme B^+^ T cells. In general, the frequencies of these cells were higher after homologous prime/boost regimens with the respective vectors than after heterologous vaccination (**Supplementary Figure S4**).

### 3.3 Heterologous prime/boost regimens with IAV-NS1 and MVA-NS1 partly protect against TBEV challenge infection

**Figure 5:**
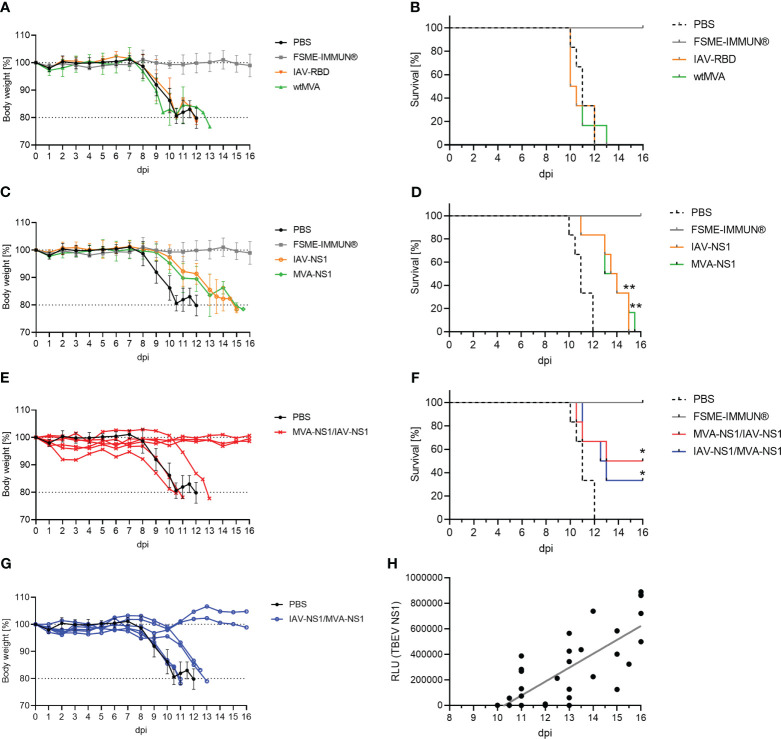
Protective efficacy of TBEV NS1-based vector constructs. Body weights and survival of BL6 mice immunized with controls **(A, B)**, IAV-NS1 or MVA-NS1 **(C, D)**, MVA-NS1/IAV-NS1 **(E, F)** or IAV-NS1/MVA-NS1 **(G, F)** after challenge infection with TBEV Neudoerfl. In **(A)** and **(C)** mean body weights are shown (n = 6). For **(E)** and **(G)** body weights from individual mice are shown. Weights of PBS mice are shown as mean (n = 6). **(B, D, F)** Kaplan-Meier curves were analyzed by log-rank test (*p<0.05, **p<0.01). **(H)** Pearson correlation of TBEV NS1-specific antibodies on day 56 before challenge infection measured by LIPS with days of survival post TBEV infection for all groups (FSME-IMMUN® group was excluded). Linear regression is depicted by gray line (r = 0.7964, p<0.0001).

Next, the protective efficacy of IAV-NS1 and MVA-NS1 immunization against a lethal challenge with TBEV Neudoerfl was assessed. All mock and vector control-vaccinated mice started to lose body weight 8 dpi and developed severe signs of TBEV infection reaching the HEP at 10-13 dpi (**Figures 5A, B**). All FSME-IMMUN®-vaccinated mice, which served as positive controls, survived until the SEP (16 dpi) without severe clinical scores (**Figures 5A, B**).

Mice that had received two doses of either IAV-NS1 or MVA-NS1 displayed a delayed onset of body weight loss starting at 10 dpi and a statistically significant prolonged survival up to 15 dpi compared to the PBS group (**Figures 5C, D**). Interestingly, 50% of the MVA-NS1/IAV-NS1- (3/6) and 33% of the IAV-NS1/MVA-NS1- (2/6) vaccinated mice were fully protected against lethal TBEV challenge (**Figures 5E–G**). These mice maintained their body weight and did not show clinical signs until SEP. Of note, prolonged survival post challenge infection correlated with levels of NS1-specific antibodies prior to infection (r=0.7964; p<0.0001) (**Figure 5H**).

### 3.4 Heterologous prime/boost vaccination reduces viral loads and histopathological changes in the central nervous system and intestine

**Figure 6:**
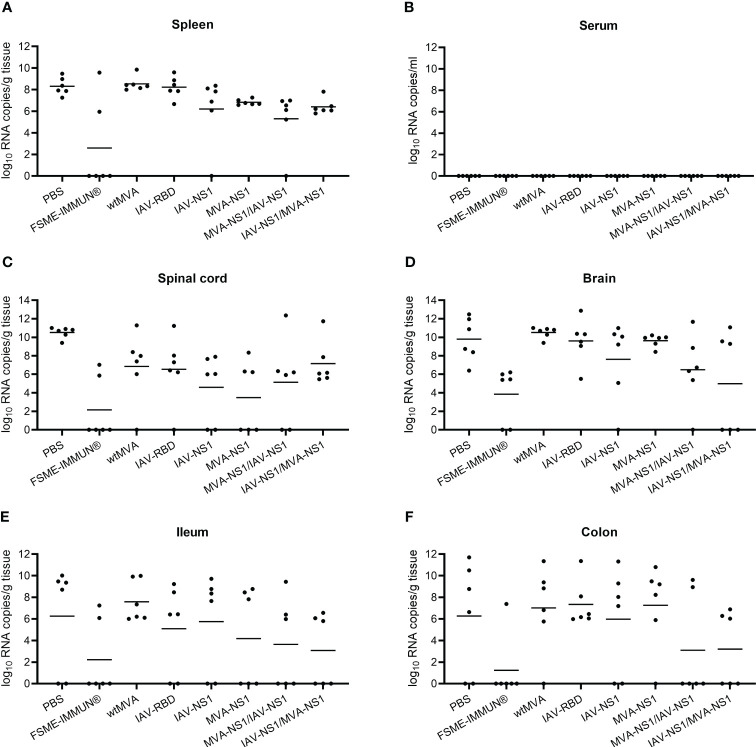
Viral loads in the periphery, CNS and GIT. Presence of TBEV RNA in spleen **(A)**, serum **(B)**, spinal cord **(C)**, brain **(D)**, ileum **(E)** and colon **(F)** was determined by performing real time RT-qPCR on cleared organ homogenates or serum from TBEV challenged mice sacrificed at 8 dpi (n = 6). Bars depict geometric means.

To assess whether NS1 vaccination against TBEV has an effect on viral replication, half of the mice from each group (n = 6) were taken out of the challenge experiment 8 dpi to examine TBEV titers in different organs by TCID_50_ and TBEV RNA copy numbers by real time RT-qPCR. At 8 dpi, high viral loads were observed in the spleens of mock-vaccinated mice with RNA copy numbers of around 10^8^ per gram of tissue, which were similar in the vector control groups. FSME-IMMUN® vaccination afforded partial protection and prevented virus replication in 4 out of 6 mice (**Figure 6A**). Interestingly, vaccination with MVA-NS1/IAV-NS1 or IAV-NS1/MVA-NS1 reduced the virus loads in the spleen approximately 100-fold compared to mock-vaccinated mice. None of the serum samples were tested positive for viral RNA 8 dpi (**Figure 6B**). Except for one FSME-IMMUN®-vaccinated mouse, no infectious virus was seen in any spleen and serum samples (**Supplementary Figures S5A, B**).

**Figure 7:**
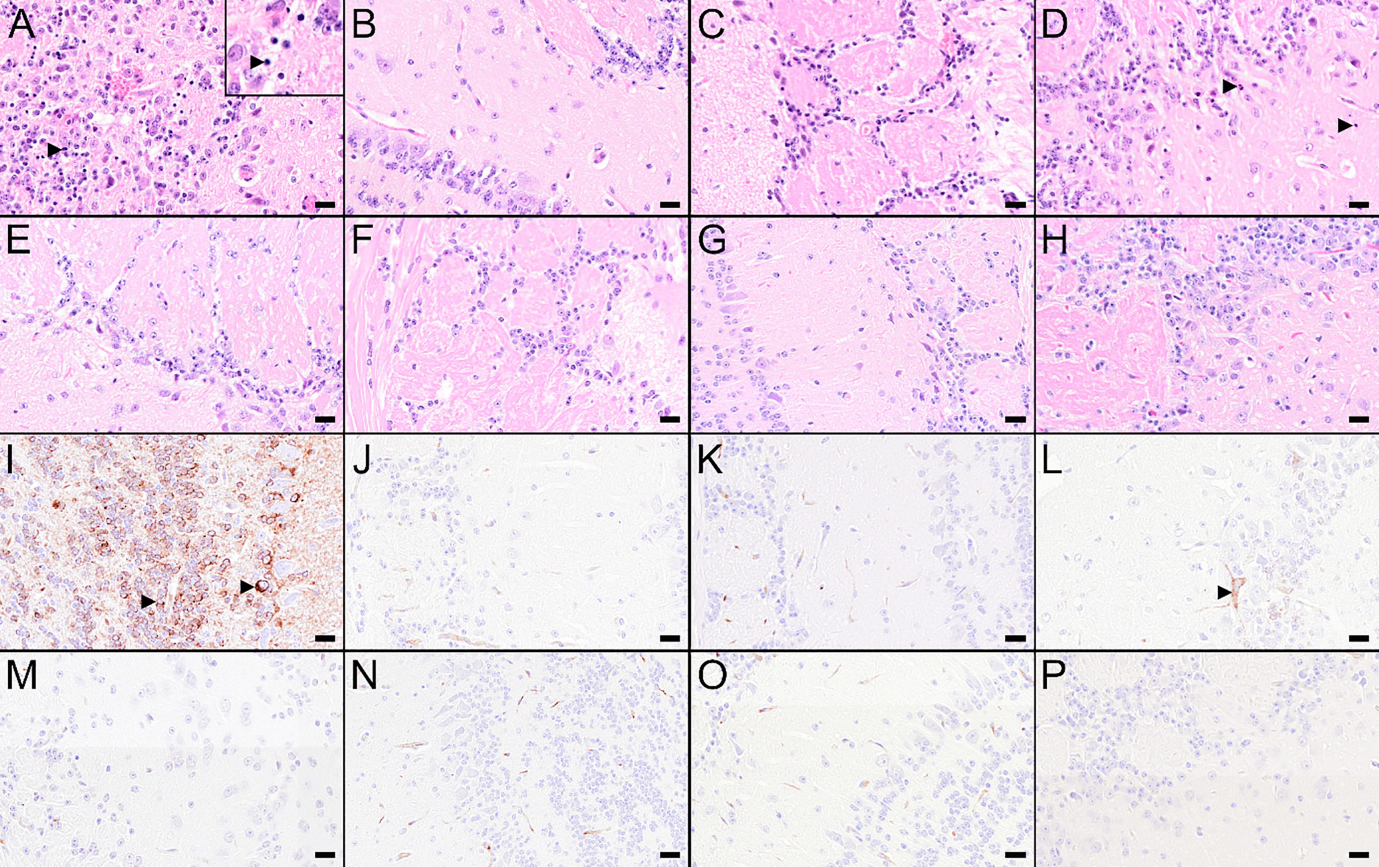
Histological and immunohistochemical changes in the olfactory bulb at 8 dpi. **(A–H)** Hematoxylin and eosin (H&E) stained sections of the olfactory bulb of a mouse treated with PBS **(A)** or vaccinated with FSME-IMMUN® **(B)**, wtMVA **(C)**, IAV-RBD **(D)**, IAV-NS1 **(E)**, MVA-NS1 **(F)**, MVA-NS1/IAV-NS1 **(G)** or IAV-NS1/MVA-NS1 **(H)**, respectively. **(A)** Olfactory bulb of the PBS-treated mouse displays marked cellular necrosis with karyorrhectic, karyolytic and pyknotic cells (insert, arrowhead) and shrunken, hypereosinophilic, triangular shaped necrotic neurons as well as inflammatory cell infiltrates. **(B–H)** In FSME-IMMUN®- **(B)**, wtMVA- **(C)**, IAV-NS1- **(E)**, MVA-NS1- **(F)**, MVA-NS1/IAV-NS1- **(G)** or IAV-NS1/MVA-NS1- **(H)** vaccinated mice, no significant microscopic lesions within the olfactory bulb are visible. In the IAV-RBD **(D)** vaccinated mouse, single necrotic cells are present. **(I–P)** Immunohistochemistry for TBEV E antigen of the olfactory bulb of a mouse treated with PBS **(I)** or vaccinated with FSME-IMMUN® **(J)**, wtMVA **(K)**, IAV-RBD **(L)**, IAV-NS1 **(M)**, MVA-NS1 **(N)**, MVA-NS1/IAV-NS1 **(O)** or IAV-NS1/MVA-NS1 **(P)**, respectively. **(I)** Immunohistochemically, a cytoplasmic TBEV immunoreactivity (arrowhead) is present in multiple cells of the olfactory bulb from the PBS-treated mouse. **(J–P)** Olfactory bulbs of FSME-IMMUN®- **(J)**, wtMVA- **(K)**, IAV-NS1- **(M)**, MVA-NS1- **(N)**, MVA-NS1/IAV-NS1- **(O)** or IAV-NS1/MVA-NS1- **(P)** vaccinated mice do not show immunoreactivity for TBEV, while the IAV-RBD- **(N)** vaccinated mouse shows single TBEV-positive cells (arrowhead). Scale bars: 20µm.

To assess the viral spread into the central nervous system (CNS), the cervical part of the spinal cord and brain tissues were tested for the presence of virus. Additionally, the olfactory bulbs of selected mice were histologically and immunohistochemically analyzed (**Figure 7**). In the spinal cords and brains of mock-vaccinated mice, high TBEV RNA copy numbers were detected 8 dpi (**Figures 6C, D**). In the brains of these mice also high titers of infectious virus were detected (**Supplementary Figure S5D**). This was accompanied by microscopic lesions in the brains of these mice consisting of cellular necrosis, microgliosis, perivascular inflammation and vasculitis 8 dpi. Cellular necrosis was characterized by shrunken and hypereosinophilic cells with karyorrhectic, karyolytic and pyknotic nuclei. Shrunken, hypereosinophilic, triangular shaped necrotic neurons are shown representatively for the olfactory bulb of a selected mock-vaccinated mouse (**Figure 7A**). Accordingly, IHC for TBEV E protein revealed high numbers of antigen-positive cells characterized by a cytoplasmic staining as shown for the olfactory bulb in **Figure 7I**.

Vaccination with FSME-IMMUN® reduced TBEV replication considerably, but not completely. In 2 and 4 out of six mice low levels of viral RNA were still detectable in spinal cords and brains, respectively (**Figures 6C, D**). This is in accordance with findings of histological and immunohistochemical analysis (**Figures 7B, J**).

Of special interest, in mice that received heterologous prime/boost regimens MVA-NS1/IAV-NS1 or IAV-NS1/MVA-NS1, a clear reduction in viral titers (**Supplementary Figure S5D**) and RNA copy numbers (**Figure 6D**) was observed in the brains compared to mock or vector control-vaccinated mice. In the brains of these mice no major histopathological changes nor TBEV antigen positive cells using IHC were observed (**Figures 7G, H, O, P**). Mice that received two doses of IAV-NS1 or MVA-NS1, respectively, showed some reduction in viral loads in the CNS (**Figures 6C, D**, **Supplementary Figures S5C, D**). However, the histopathological changes in the CNS of these mice were less prominent than in the CNS of mock-vaccinated animals (**Figures 7E, F, M, N**).

Upon dissection at 8 dpi, 100% of the mock-vaccinated and 67% of the vector control mice displayed macroscopically visible acute distension and segmental dilation of the gastrointestinal tract as described previously (54). In contrast, only 50% of mice vaccinated with two doses of IAV-NS1 or MVA-NS1 displayed such lesions. In mice that received the heterologous prime/boost vaccination regimens, this proportion was only 16.7%. No macroscopic abnormalities were observed in FSME-IMMUN®-vaccinated mice (data not shown).

In the majority of the mock and vector control-vaccinated mice, high TBEV RNA levels were observed in ileum and colon at 8 dpi with RNA copy numbers ranging from 10^6^-10^12^ per gram of tissue (**Figures 6E, F**). Infectious virus was mainly detectable in the colon of infected animals (**Supplementary Figures S5E, F**). In FSME-IMMUN®-vaccinated mice, geometric mean titers were considerably reduced and viral RNA was detected in two and one animals in ileum and colon, respectively (**Figures 6E, F**). In these mice infectious virus was not detected in any of the organs tested 8 dpi (**Supplementary Figures S5E, F**) or 16 dpi (data not shown). Of interest, also in mice that received MVA-NS1/IAV-NS1 or IAV-NS1/MVA-NS1 viral copy numbers, especially in the colon, were reduced >1000-fold compared to mock and vector control-vaccinated mice (**Figures 6E, F**).

**Figure 8:**
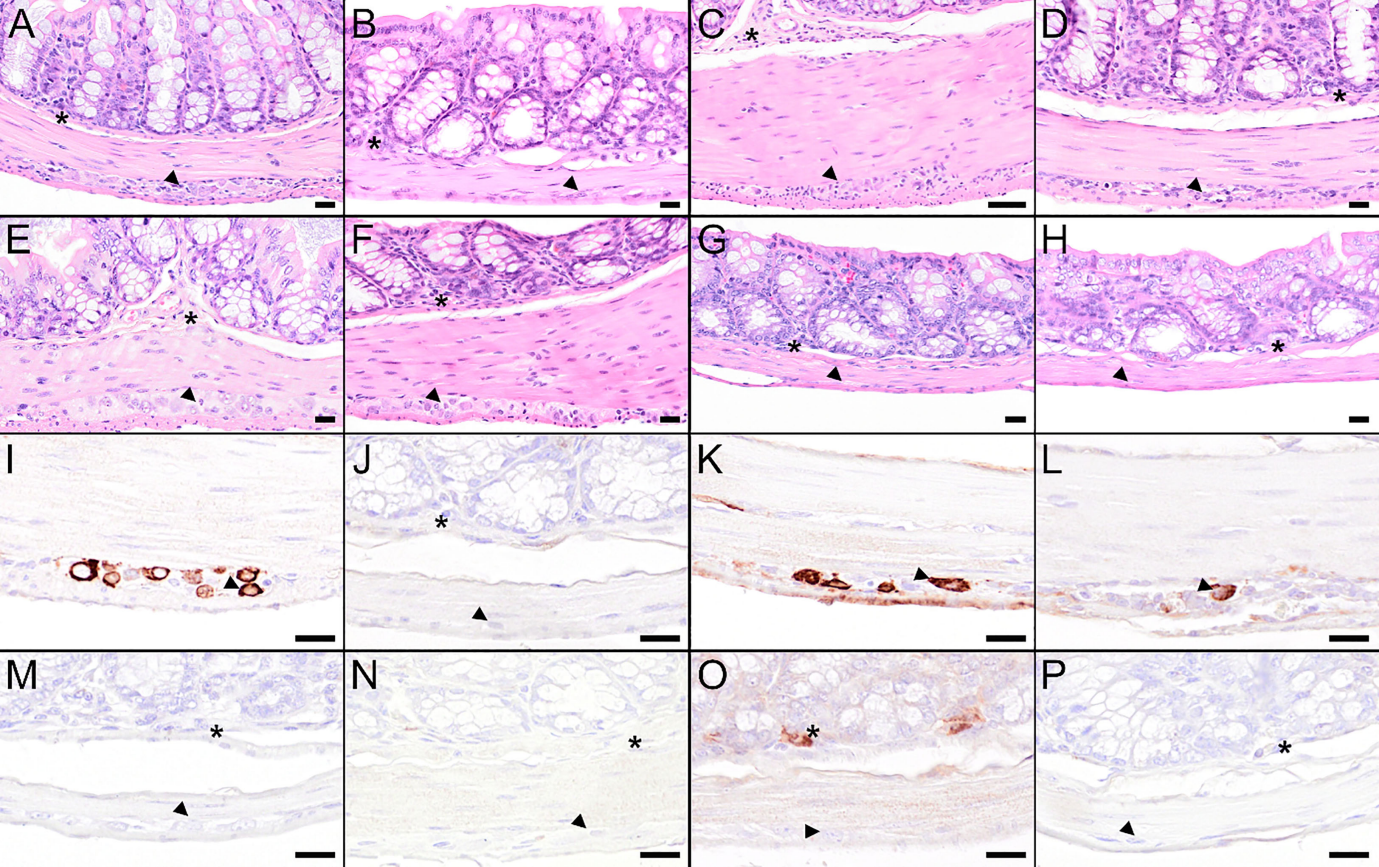
Histological and immunohistochemical analysis of colon at 8 dpi. **(A–H)** Hematoxylin and eosin (H&E) stained sections of the colon of a mouse treated with PBS **(A)** or vaccinated with FSME-IMMUN®, **(B)**, wtMVA **(C)**, IAV-RBD **(D)**, IAV-NS1 **(E)**, MVA-NS1 **(F)**, MVA-NS1/IAV-NS1 **(G)** or IAV-NS1/MVA-NS1 **(H)**, respectively. **(A)** Colon of the PBS-treated mouse displays minor to mild hypercellularity and cellular necrosis in the myenteric (arrowhead) and submucosal plexus (asterisk). **(B–F)** Colon of the FSME-IMMUN®- **(B)**, wtMVA- **(C)**, IAV-RBD- **(D)**, and MVA-NS1- **(F)** vaccinated mice show minimal to moderate histopathological changes mainly characterized by hypercellularity/inflammatory cell infiltrates in the myenteric and submucosal plexus. **(E–H)** In the IAV-NS1- **(E)**, MVA-NS1/IAV-NS1- **(G)** and IAV-NS1/MVA-NS1- **(H)** vaccinated mice, no histopathological changes in the plexi of the colon are observed. **(I–P)** Immunohistochemistry for TBEV E antigen of the colon of a mouse treated with PBS **(I)** or vaccinated with FSME-IMMUN® **(J)**, wtMVA **(K)**, IAV-RBD **(L)**, IAV-NS1 **(M)**, MVA-NS1 **(N)**, MVA-NS1/IAV-NS1 **(O)** or IAV-NS1/MVA-NS1 **(P)**, respectively. **(I, K, L)** Immunohistochemically, a cytoplasmic TBEV immunoreactivity is present in cells of the myenteric plexus (arrowhead) of the colon from the PBS-treated mouse **(I)**, the wtMVA-vaccinated mouse **(K)** and the IAV-RBD-vaccinated mouse **(L)**. A single TBEV-antigen positive cell is visible in the submucosal plexus of the colon from the MVA-NS1/IAV-NS1-vaccinated mouse (**O**, asterisk). No TBEV-antigen positive cells are detectable in the submucosal (asterisk) or myenteric plexus (arrowhead) of FSME-IMMUN®- **(J)**, IAV-NS1- **(M)**, MVA-NS1- **(N)** and IAV-NS1/MVA-NS1-vaccinated **(P)** mice. Scale bars **(A, B, D–H)**: 20µm; Scale bar: **(C, I–P)**: 50µm.

Histopathological examination of the intestine revealed histopathological lesions characterized by ganglioneuritis of the myenteric and submucosal plexus in mock and vector control-vaccinated mice 8 dpi as described previously (54). Ganglioneuritis of the myenteric and submucosal plexus are representatively shown in the colon (**Figure 8**). Ganglia show signs of neuronal necrosis and an infiltration with inflammatory cells and/or hyperplasia of resident immune cells. In concordance with the viral loads that were measured, only minor histopathological changes were observed in mice that received heterologous prime/boost NS1 vaccination (**Figures 8G, H, O, P**). In line with the low numbers of mice with infectious virus in the intestine (**Supplementary Figure S5F**), IHC showed no significant portion of antigen-positive cells in the plexus of FSME-IMMUN®- or NS1-immunized mice (**Figures 8J, M–P**), whereas TBEV antigen positive cells were shown for mock and vector control-vaccinated mice (**Figures 8I, K, L**).

For 3/5 of the surviving mice receiving the heterologous NS1-based vaccinations, neither infectious virus (data not shown) nor virus RNA were detectable in all tested organs at study endpoint (**Supplementary Figure S6**). The other two mice showed low TBEV RNA copy numbers in brain (10^5^ RNA copies/g tissue) and colon (10^6^ RNA copies/g tissue) (MVA-NS1/IAV-NS1) or colon only (IAV-NS1/MVA-NS1). Histopathological examination and IHC for TBEV of brain and intestine showed no major lesions or TBEV antigen staining in those mice as shown for representative mice and organ sections (**Supplementary Figure S7**).

## 4 Discussion

In the present study, we tested the efficacy of two TBEV NS1-carrying viral vectors to induce protective immunity against TBEV. We showed that the use of IAV- and MVA-based viral vectors were highly immunogenic, especially in heterologous prime/boost regimens, resulting in the induction of TBEV NS1-specific antibodies and T cells. The vector-induced NS1-specific immunity afforded mice partial protection against a lethal challenge infection with TBEV.

First, the two recombinant vectors were characterized *in vitro* and it was shown that they both drive the expression of TBEV NS1 intracellularly. However, cell surface expression was only observed with IAV-NS1. Since TBEV NS1 lacks a transmembrane domain, it interacts with the plasma membrane via hydrophobic protrusions and is associated with lipid rafts [reviewed in (55)]. However, in the IAV-NS1 construct, NS1 was fused to the N-terminal part of the IAV NA which contains its transmembrane domain facilitating insertion of the NA-NS1 fusion in the cell membrane and expression on the cell surface. This was further supported by the fact that NS1 secretion was only observed from cells infected with MVA-NS1 but not from cells infected with IAV-NS1 (data not shown).

Insertion of the NS1 gene did not affect the attenuated phenotype of both recombinant vectors. In the absence of exogenous NA, NA-deficient IAV-RBD and IAV-NS1 replicated only to low titers, which is in concordance with previous studies (56, 57). Attenuation is achieved by the replacement of a large part of the IAV NA, which as receptor destroying enzyme plays an important role in the IAV replication cycle and release of newly budded IAV virions. Lack of a functional NA results in aggregation of virions at the cell surface as demonstrated by electron microscopy for a NA-deficient IAV expressing a NA-GFP fusion protein (56). As shown previously, trans-complementation by addition of NA from *V. cholerae* restored the replicative capacity of NA-deficient IAVs (58). Attenuation of MVA was achieved by extensive passaging in avian cells, resulting in the loss of genes involved in virus-host interactions and replication-deficiency in most mammalian cells [reviewed in (34)]. Due to their highly attenuated phenotype both IAV-NS1 and MVA-NS1 were well tolerated by mice.

Upon vaccination with the NS1-expressing vectors, NS1-specific antibodies were readily induced in all mice. As expected, these antibodies fail to display neutralizing activity because NS1 is not a component of TBEV virions. In contrast, vaccination with FSME-IMMUN®, which was included as a positive control in our study design, induced high levels of VN antibodies but failed to induce TBEV NS1-specific antibodies. These findings are in line with a recent publication showing that a high number of FSME-IMMUN® vaccine doses is required to induce measurable quantities of NS1-specific antibodies in mice (18). Two doses of MVA-NS1 proved to be more immunogenic than two doses of IAV-NS1. MVA and IAV are two different viral expression systems with their own unique biological properties. Thus, the level of NS1 gene expression from these viral vectors may differ. Furthermore, as mentioned above, NS1 was secreted from MVA-NS1-infected cells, whereas IAV-NS1 drove the expression of a membrane NA-NS1 fusion protein that was not secreted. To increase NS1 expression and immunogenicity by the IAV vector, the exchange of 5´- and 3´-end NA packaging signals with those of the HA gene might be considered (59). For both IAV-NS1 and MVA-NS1, the respective homologous booster vaccination significantly increased NS1-specific antibody levels induced after the first immunization. This indicates that vector-immunity induced by the prime immunization did not prevent boosting of the NS1-specific antibody response as has been shown previously (60–63). It is well known that prime/boost vaccination regimens with heterologous vectors or antigen delivery systems can improve immune responses significantly [(64–66), reviewed in (35)]. The use of IAV-NS1 and MVA-NS1 for heterologous prime/boost vaccination increased the NS1-specific antibody and T cell responses significantly compared to prime/boost with the same vectors. The order of immunization with the two vectors did not make a big difference, which is in contrast to a previous vaccination study with recombinant IAV and VACV expressing malaria antigens (67). The superior immunogenicity of heterologous prime/boost vaccination with IAV-NS1 or MVA-NS1 translated also in superior protection of mice against infection with TBEV Neudoerfl. In comparison to PBS and vector control groups, a delayed onset of body weight loss and significant prolonged survival was observed in mice that received two doses of the same vector. No difference in disease progression and survival between IAV-NS1 and MVA-NS1 immunized mice was observed, although MVA-NS1 was more immunogenic. In contrast, heterologous prime/boost vaccination resulted in partial survival of 33% and 50% of mice vaccinated with IAV-NS1/MVA-NS1 or MVA-NS1/IAV-NS1, respectively. In general, these results are in agreement with previous studies which demonstrated that with NS1-based vaccine preparations partial protection against TBEV infection can be achieved (21–27). Reduced viral loads in the periphery, CNS and intestine suggest that induction of immunity to NS1 favors restriction of virus replication and associated pathological changes in infected tissues.

Although the exact mode of protective immunity is unclear, adoptive transfer experiments with TBEV NS1-specific serum or B cells indicated that antibodies are involved as a correlate of protection (22, 25). Also in our study, the magnitude of the NS1-specific antibody response correlated with the duration of survival. Several studies identified antibody-dependent complement-mediated cytolysis (CMC) as a possible mechanism involved in protection [reviewed in (19)]. However, NS1-immunized complement-deficient mice were still protected upon TBEV challenge infection indicating that protective immunity is not exclusively mediated by CMC (21). As shown for other flaviviruses, NS1-specific antibodies can also contribute to complement-independent Fc-mediated effector functions like antibody-dependent cellular cytotoxicity and antibody-dependent cellular phagocytosis (68–73). Therefore, the mechanism of how TBEV NS1-specific antibodies exert their protective effect should be subject of further investigation. Since the homology of the NS1 amino acid sequence of European and Far-Eastern TBEV subtypes is between 92-98.9% (74) and cross-reactivity of NS1-specific antibodies was shown between these subtypes (20), we speculate that NS1-specific immunity offers a certain degree of cross-protection against other TBEV subtypes.

The use of NS1 as vaccine antigen may offer the advantage over the use of the E protein that NS1 vaccination will not result in the induction of virion-reactive antibodies. Consequently, the risk of ADE of infection is reduced (28). Although clear evidence for ADE of TBEV infection *in vivo* is lacking [reviewed in (15)], ADE was shown *in vitro* (29–32). Furthermore, flavivirus NS1, like that of Dengue virus (DENV), may contribute to pathogenesis by causing plasma leakage, thrombocytopenia and hemorrhages, all characteristic of severe dengue disease. Moreover, autoantibodies elicited by DENV NS1 can cross-react with host-antigens on e.g. endothelial cells or coagulation factors by molecular mimicry [reviewed in (75, 76)]. In our studies, we did not observe any obvious detrimental effects of TBEV NS1 expression or NS1-specific antibodies in mice. NS1-immunized animals developing TBE displayed no signs of increased TBE severity compared to infected control mice. NS1 of the respective flaviviruses may play distinct roles during infection and in pathogenesis which is underscored by the low sequence homology (37%) between TBEV and DENV NS1 (25).

Apart from NS1-specific antibodies, NS1-specific T cells can contribute to protective immunity against TBEV infection as was demonstrated in early adoptive transfer experiments (22). For Zika virus (ZIKV) it was shown that functional CD4^+^ and CD8^+^ T cell responses were required to control and clear infection in ZIKV NS1-vaccinated mice, despite the presence of high anti-NS1 antibody levels (77). In our study, mice that received a heterologous prime/boost vaccination had stronger NS1-specific IFN-γ^+^ T cell responses than those received two doses of the same viral vector. CD4^+^ and CD8^+^ T cells contributed to this response of which the CD8^+^ T cells also were Granzyme B positive. The induction of these improved T cell responses may have been the basis for the partial protection against TBEV challenge infection. In addition to Fc-mediated viral clearance of infected cells via NS1-specific antibodies, CD4^+^ and CD8^+^ cytotoxic T cells may have contributed to restricted viral replication in spleen, CNS and intestine in mice that received heterologous prime/boost vaccination. Although it has been demonstrated in TBEV mouse models that CD8^+^ T cells can have detrimental effects and contribute to the pathogenesis of infection (78), we did not observe such effects. Examination of representative H&E stained tissue sections of the CNS and intestine did not reveal more severe histopathological changes in mice that received the heterologous prime/boost vaccination than in those that received two doses of the same vector. However, to obtain a better understanding of the role of TBEV NS1-specific T cells in the pathogenesis of TBEV infection, further studies are required like adoptive T cell transfer experiments with selected T cell populations obtained from NS1-vaccinated mice.

In conclusion, we have shown that with the viral vectors IAV-NS1 and MVA-NS1 potent NS1-specific antibody and T cell responses can be induced in mice using heterologous prime/boost vaccination regimens. This NS1-specific immunity afforded partial protection against a lethal challenge infection with the Neudoerfl strain of TBEV. Therefore, the inclusion of a NS1 component in improved next generation TBEV vaccines seems desirable as has been demonstrated for other flaviviruses, like Japanese encephalitis virus and ZIKV (79, 80). Ideally, in such vaccines NS1 would be combined with the E or prM/E proteins, the major target for the induction of VN antibodies.

## Data availability statement

The raw data supporting the conclusions of this article will be made available by the authors, without undue reservation.

## Ethics statement

The animal study was reviewed and approved by Lower Saxony State Office for Consumer Protection and Food Safety (LAVES, Approval No. 33.8-42502-04-20/3437).

## Author contributions

Conceptualization: JBe, MK, TG, CKP, and GR. Formal analysis: JBe and MK. Investigation: JBe, MK, JBi, and CKP performed the animal experiments and analyzed the samples. IZ, CP, and WB performed and analyzed histology and immunohistochemistry data. Resources: GS. Writing-original draft preparation: JBe and MK. Writing-review and editing: IZ, CP, JBi, TG, WB, AO, GS, CKP, and GR. Visualization: JBe and MK. Supervision: TG, CKP, and GR. Funding acquisition: GR. All authors contributed to the article and approved the submitted version.
