# Supplementary material for: Induction of humoral and cell-mediated immunity to the NS1 protein of TBEV with recombinant Influenza virus and MVA affords partial protection against lethal TBEV infection in mice
Source: Front Immunol. 2023 Jul 7;14:1177324. doi: 10.3389/fimmu.2023.1177324 (PMC10360051; doi:10.3389/fimmu.2023.1177324)
Supplement: Supplementary file 1 [file DataSheet_1.docx]

Supplementary Material

Induction of humoral and cell-mediated immunity to the NS1 protein of TBEV with recombinant influenza virus and MVA affords partial protection against lethal TBEV infection in mice

# Supplementary Figures


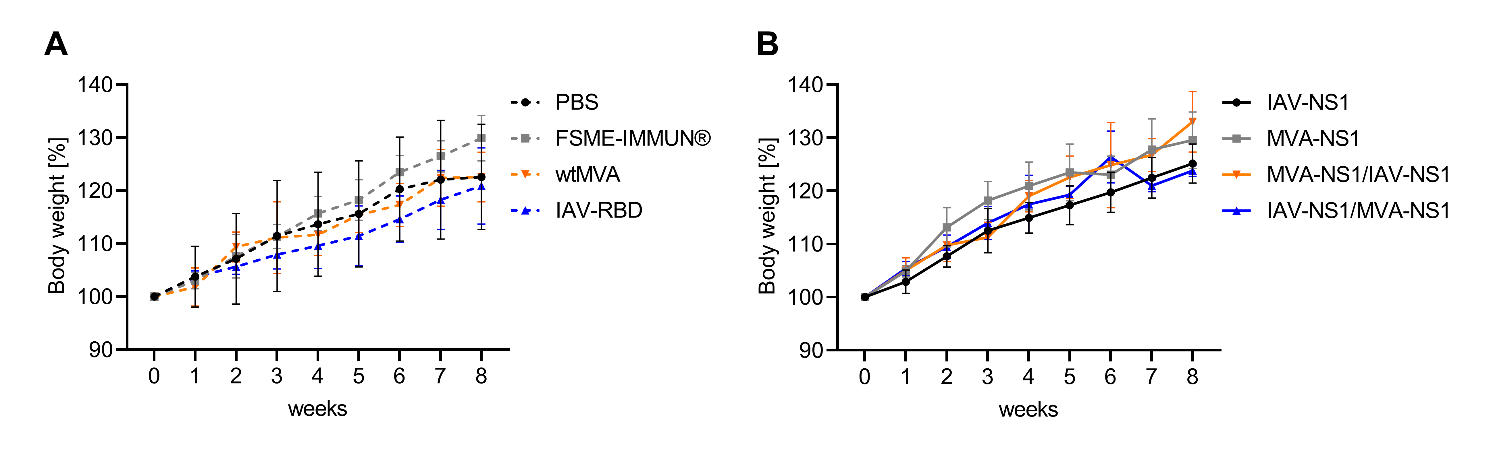


Supplementary Figure S1. **Body weights upon immunization**. Body weight curves of mice immunized twice in a 4-week interval with (A) PBS (dotted black), FSME-IMMUN® (dotted grey) or vector controls (wtMVA (dotted orange) or IAV-RBD (dotted blue)) or (B) IAV-NS1 (black), MVA-NS1 (grey), MVA-NS1/IAV-NS1 (orange) or IAV-NS1/MVA-NS1 (blue). Mean values with SD are shown (n=4 mice/group).


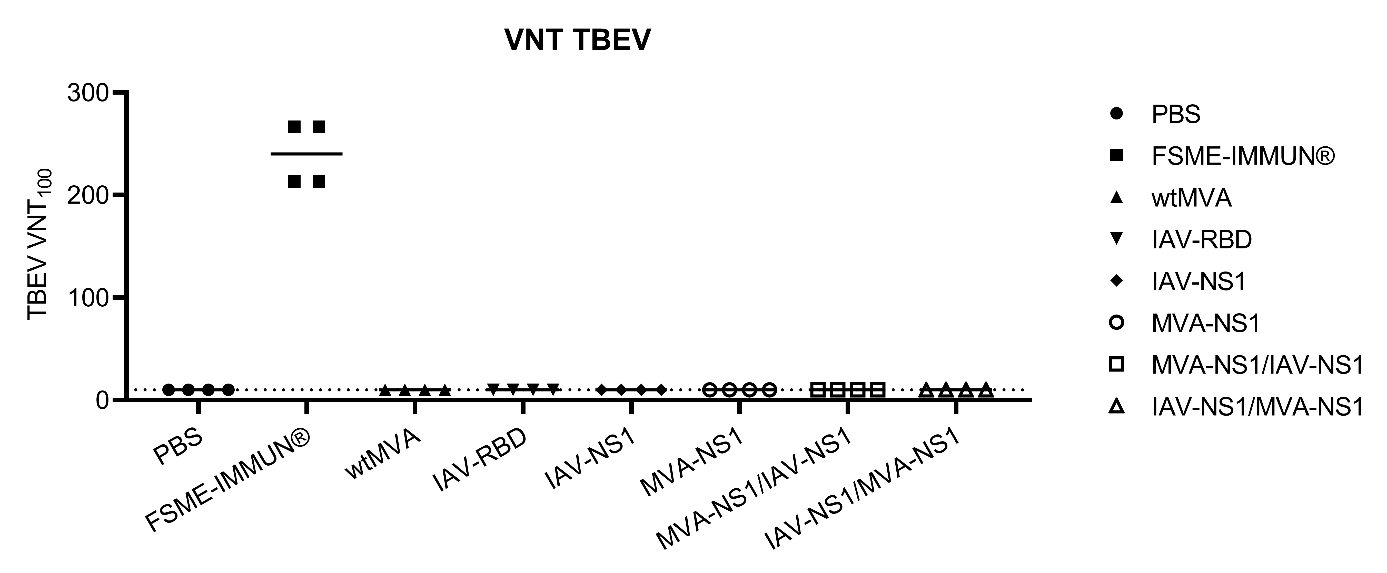


Supplementary Figure S2. **VN antibodies against TBEV**. TBEV-specific neutralizing antibodies four weeks after boost vaccination were measured by performing VN assay with 100 TCID_50_ TBEV Neudoerfl in triplicates on A549 cells. Serum starting dilution was 1:10 resulting in detection limit of 10 (dotted line).


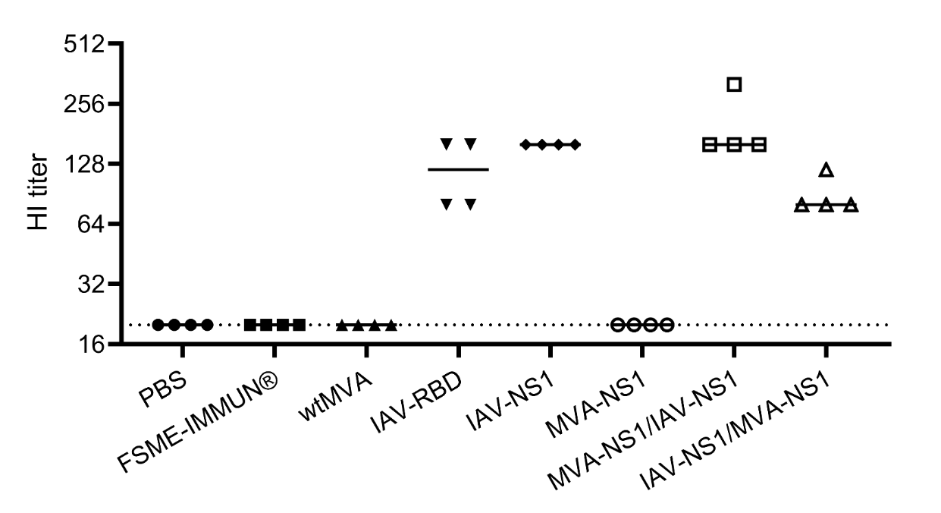


Supplementary Figure S3. **Antibody response against the IAV-vector**. HI titers against rPR8 assayed with 1% chicken red blood cells and 4 HAU rPR8. Detection limit is indicated by the dotted line. Median values are represented by black bars.


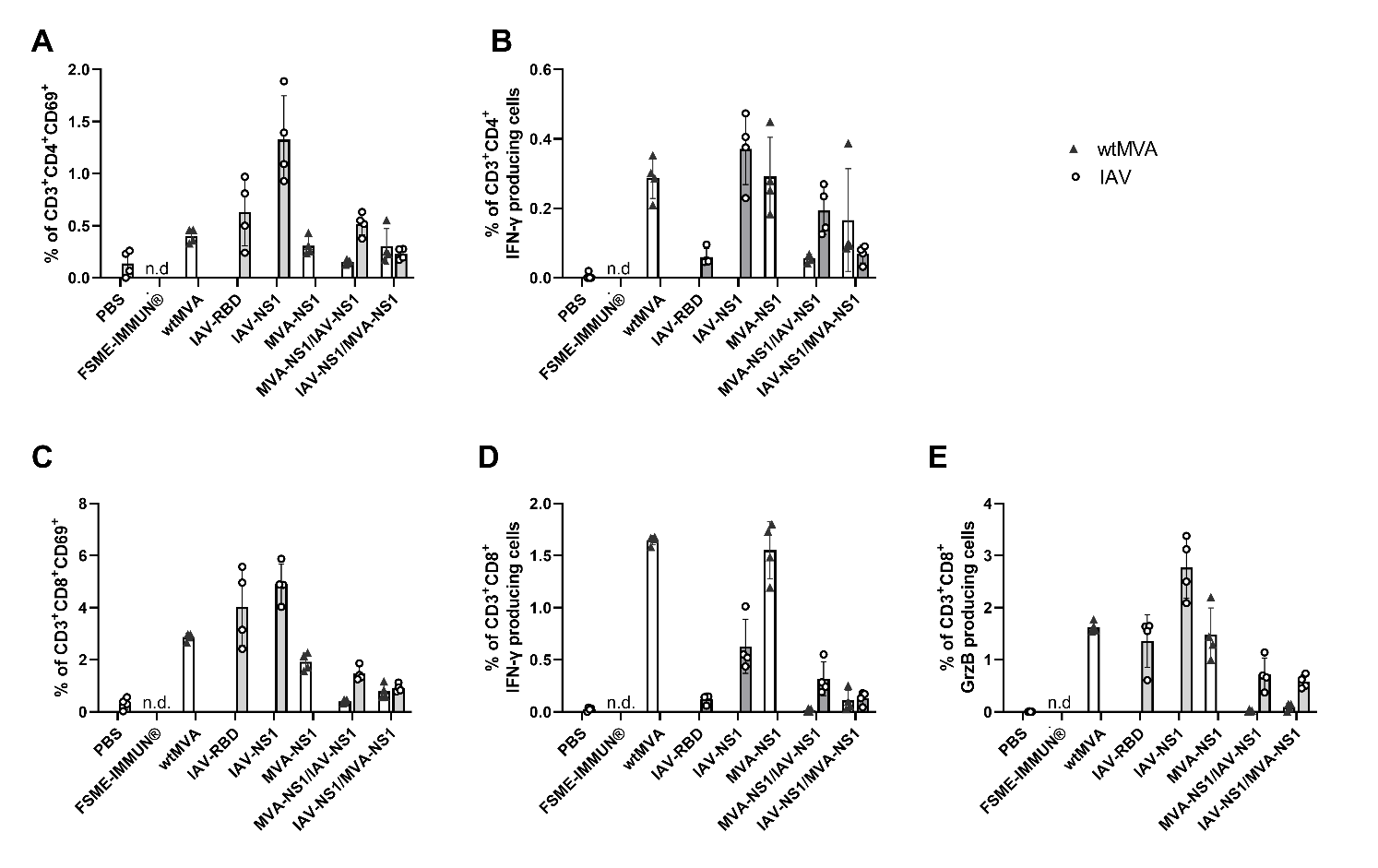


Supplementary Figure S4. **T cell response against vectors analyzed by FACS**. Frequency of CD3^+^ subpopulations gated on CD4^+^ (A, B) and CD8^+^ (C, D, E) T cells positive for CD69, IFN-γ and Granzyme B (GrzB) upon restimulation with live wtMVA (triangle) or IAV (unfilled circle) (n = 4). Bars represent mean with SD. Data is shown after background subtraction. N.d. = not determined.


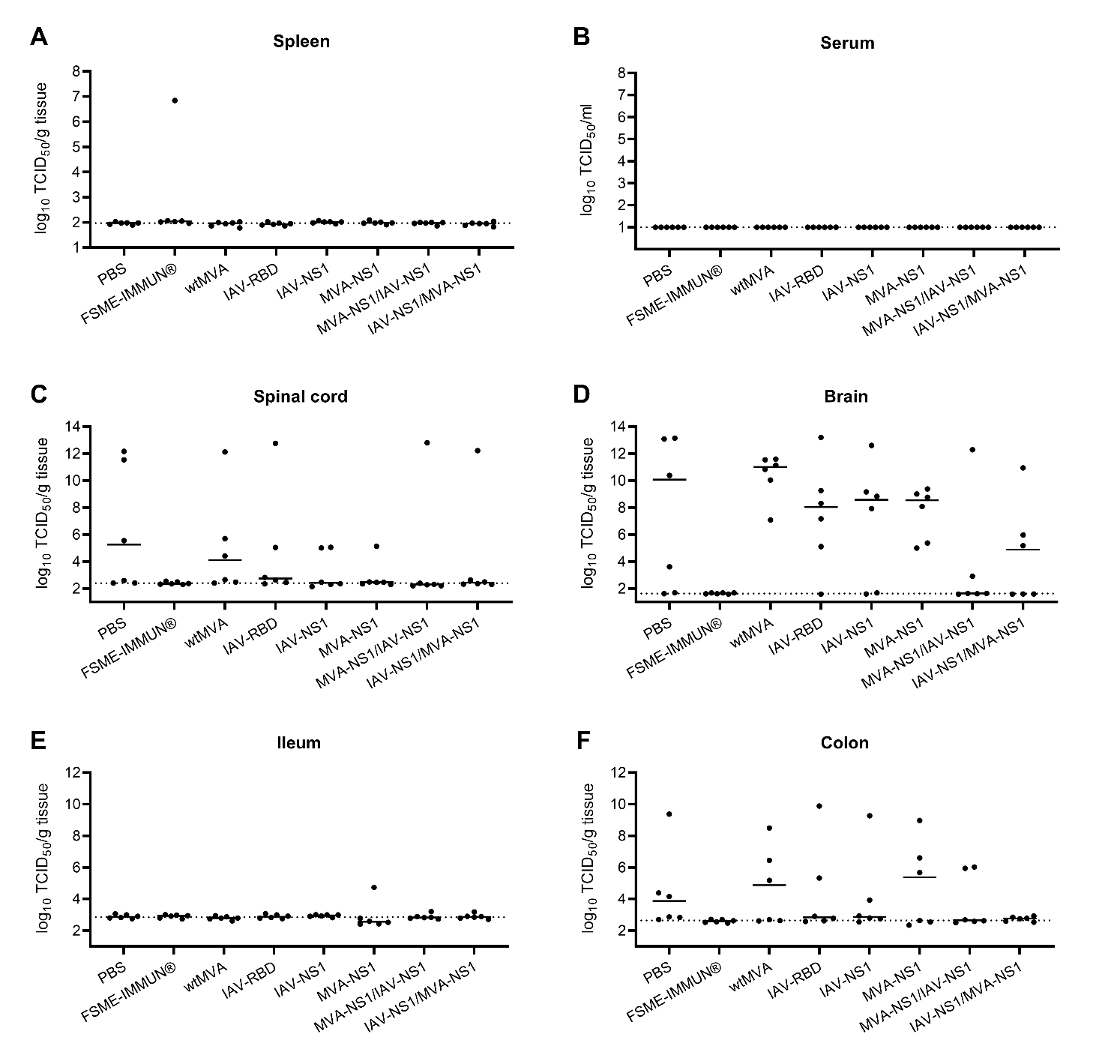


Supplementary Figure S5. **Infectious virus in the periphery, CNS and intestine 8 dpi**. Presence of infectious virus was detected by inoculation of A549 cells with 1:10 serial dilutions of serum (B) or cleared organ homogenates from spleens (A), cervical part of spinal cord (C), brain (D), ileum (E) and colon (F) of vaccinated and TBEV infected mice sacrificed 8 dpi (n = 6). Virus titers were determined by CPE-based read-out and calculated to TCID50 per gram tissue or per ml. Dotted lines indicate detection limits. Median values are shown in all graphs.


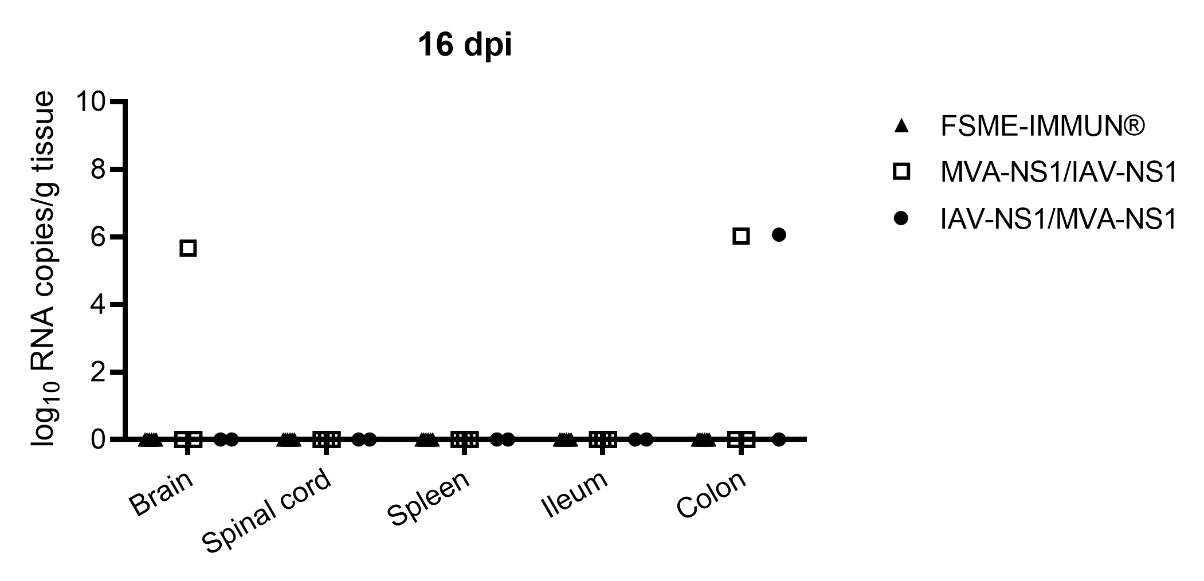


Supplementary Figure S6. **TBEV RNA copy numbers in surviving mice 16 dpi**. Presence of TBEV RNA in different organs was determined by performing real time RT-qPCR on cleared organ homogenates from TBEV challenged mice sacrificed 16 dpi (FSME-IMMUN® n = 6, MVA-NS1/IAV-NS1 n = 3, IAV-NS1/MVA-NS1 n = 2).


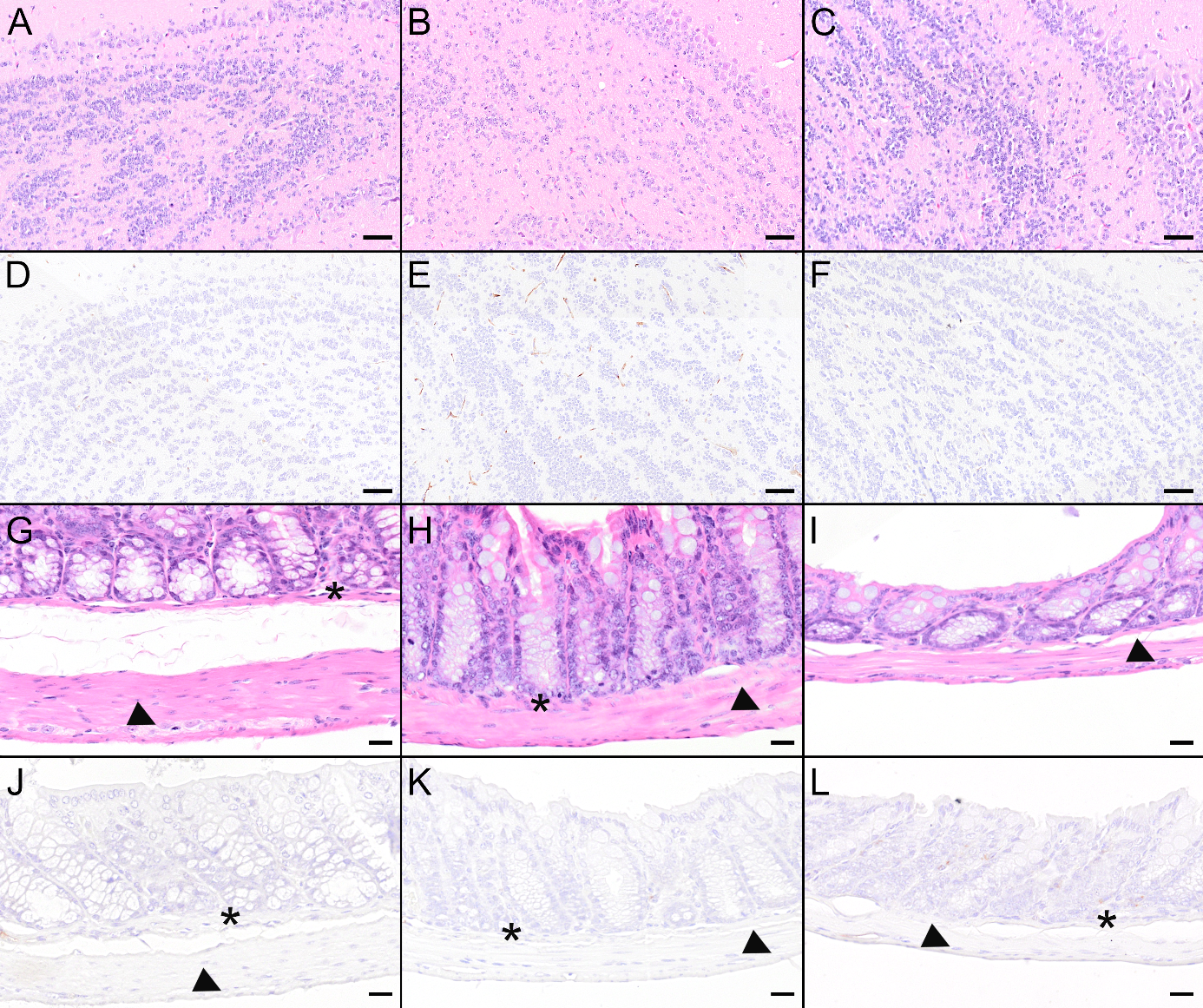


Supplementary Figure S7. **Histological and immunohistochemical analysis of olfactory bulb and colon of surviving mice 16 dpi.** (A-F) Hematoxylin and eosin stained sections (A-C) and immunohistochemistry for TBEV E protein (D-F) of olfactory bulb from a mouse vaccinated with FSME-IMMUN® (A, D), MVA-NS1/IAV-NS1 (B, E) or IAV-NS1/MVA-NS1 (C, F), respectively. There are no significant microscopic lesions within the olfactory bulb of all three mice and no immunoreactivity for TBEV E protein is visible. (G-L) Hematoxylin and eosin stained sections (G-I) and immunohistochemistry for TBEV E protein (J-L) of colon from a mouse vaccinated with FSME-IMMUN® (G, J), MVA-NS1/IAV-NS1 (H, K) or IAV-NS1/MVA-NS1 (I, L), respectively. There are no significant microscopic lesions within the myenteric (arrowhead) or submucosal (asterisk) plexus of the colon of all three mice and no positive staining for TBEV E protein is present. Scale bars: 50µm.
